# Genome-wide effects of MELK-inhibitor in triple-negative breast cancer cells indicate context-dependent response with p53 as a key determinant

**DOI:** 10.1371/journal.pone.0172832

**Published:** 2017-02-24

**Authors:** Marisa Simon, Fahmi Mesmar, Luisa Helguero, Cecilia Williams

**Affiliations:** 1 Center for Nuclear Receptors and Cell Signaling, Department of Biology and Biochemistry, University of Houston, Texas, United States of America; 2 Institute for Research in Biomedicine, Department of Biosciences, University of Aveiro, Aveiro, Portugal; 3 Division of Proteomics, SciLifeLab, School of Biotechnology, KTH – Royal Institute of Technology, Solna, Sweden; 4 Department of Biosciences and Nutrition, Karolinska Institutet, Stockholm, Sweden; Florida International University, UNITED STATES

## Abstract

Triple-negative breast cancer (TNBC) is an aggressive, highly recurrent breast cancer subtype, affecting approximately one-fifth of all breast cancer patients. Subpopulations of treatment-resistant cancer stem cells within the tumors are considered to contribute to disease recurrence. A potential druggable target for such cells is the maternal embryonic leucine-zipper kinase (MELK). MELK expression is upregulated in mammary stem cells and in undifferentiated cancers, where it correlates with poor prognosis and potentially mediates treatment resistance. Several MELK inhibitors have been developed, of which one, OTSSP167, is currently in clinical trials. In order to better understand how MELK and its inhibition influence TNBC, we verified its anti-proliferative and apoptotic effects in claudin-low TNBC cell lines MDA-MB-231 and SUM-159 using MTS assays and/or trypan blue viability assays together with analysis of PARP cleavage. Then, using microarrays, we explored which genes were affected by OTSSP167. We demonstrate that different sets of genes are regulated in MDA-MB-231 and SUM-159, but in both cell lines genes involved in cell cycle, mitosis and protein metabolism and folding were regulated. We identified p53 (TP53) as a potential upstream regulator of the regulated genes. Using western blot we found that OTSSP167 downregulates mutant p53 in all tested TNBC cell lines (MDA-MB-231, SUM-159, and BT-549), but upregulates wild-type p53 in the luminal A subtype MCF-7 cell line. We propose that OTSSP167 might have context-dependent or off-target effects, but that one consistent mechanism of action could involve the destabilization of mutant p53.

## Introduction

Triple-negative breast cancer (TNBC) is a breast cancer (BC) subtype characterized by highly undifferentiated, aggressive, and metastatic cells. Since TNBC lacks expression of the receptors currently used for targeted treatment (ER and HER2), it is treated with conventional surgery, radiation, and chemotherapy. Although TNBC is sufficiently chemosensitive, patients with this subtype have a higher risk of recurrence within the first three years and a poorer prognosis if the cancer metastasizes [1, 2]. There is a major need for new therapeutic targets for this subtype, and several have been proposed, including poly-ADP ribose polymerase (PARP), cell cycle checkpoint proteins, and phosphoinositide 3-kinase (PI3K) pathway proteins. However, small molecule inhibitors of these targets are only effective in certain subpopulations of TNBC patients [3].

TNBC is a heterogeneous disease with several subclasses, including basal-like 1 or 2 (BL 1/2), immunomodulary (IM), mesenchymal (M), mesenchymal stem-like (MSL), luminal androgen receptor (LAR) and claudin-low [4]. Relative to other TNBC subtypes, claudin-low is characterized by a low expression of epithelial tight-junction claudin proteins, mucin 1 (MUC1), EPCAM and E-cadherin (CDH1), and high expression of epithelial-to-mesenchymal transition (EMT) markers, along with cancer stem cell (CSC) characteristics [5, 6]. It is hypothesized that the combination of these factors predisposes this TNBC population to become invasive and resistant to treatment [7, 8]. Cells with CSC characteristics are thought to re-propagate tumors after resisting conventional cancer treatment, thereby contributing to TNBC’s high rates of recurrence. Consequently, it is of specific interest to target these cells.

The maternal embryonic leucine-zipper kinase (MELK) is an interesting target for TNBC and its CSC populations. High MELK expression correlates with poor prognosis in breast cancers [9] and MELK is included in three different multi-gene expression profiles that predict BC aggressiveness, prognosis, and therapy response in the clinical setting [10]. MELK has been found to be essential for mitotic progression in TNBC [11], and we have previously shown that MELK expression is high in non-tumorigenic murine mammary stem-like cells, but disappears when the cells are induced to differentiate [12]. Additionally, in multipotent neural progenitors (MNPs), MELK is considered to be a marker of self-renewal [13] and MELK depletion sensitizes colorectal cancer cells to radiation or 5-FU treatment [14].

A competitive type I kinase inhibitor, OTSSP167 (OTS167) has been designed to inhibit MELK activity [15], and its efficacy has been explored in several cancers including in TNBC cell lines [11, 15, 16]. Several phase I clinical trials with OTS167 are in process for solid cancers, leukemia, and TNBC (clinicaltrials.gov). In the present study, we aim to better understand how this inhibitor and MELK impacts TNBC cells by exploring the genome-wide impact of OTS167 treatment in claudin-low TNBC cells, in order to begin to elucidate corresponding mechanisms and effects.

## Methods and materials

### Cell lines and culture materials

MDA-MB-231 (HTB-26), MCF-7 (HTB-22), T47D (HTB-133) and MCF10A (CRL-10317) cell lines were obtained from the American Type Culture Collection (ATCC, Rockville, MD, USA). SUM-159 and BT-549 cell lines were gifts (Melissa Landis, Houston Methodist Research Institute and Christoforos Thomas, University of Houston). Cell culture media and fetal bovine serum (FBS) were obtained from Invitrogen (Invitrogen, Carlsbad, CA, USA). MDA-MB-231 was cultured in DMEM/F-12 1:1 mixture, SUM-159 and MCF-7 in DMEM alone, and BT-549 in RPMI media. All media contained Phenol Red, 10% (v/v) FBS, and 1% (v/v) penicillin streptomycin. Cells were treated with indicated concentrations of OTS167 kinase inhibitor (Chemscene, Junction, NJ) or vehicle (DMSO).

### Proliferation assays

MTS and trypan blue exclusion assays were used to assess cell proliferation or cell survival. We used CellTiter96 AQueous One Solution (Promega, Madison, WI, USA) in quintuplet biological replicates, according to the manufacturer’s protocol. The EC_50_ of OTS167 in each cell line was calculated using the nonlinear regression (Curve Fit) function in Graph Pad Prism. Standard trypan blue exclusion assays were performed in biological triplicates after 72h treatment with OTS167, using the Countess automated cell counter (Invitrogen). The two-tailed Student's t-test was used for statistical testing, and results were considered significant if p<0.05.

### RNA extraction

Total RNA was extracted with TRIzol (Invitrogen) and purified with RNeasy spin columns (Qiagen, Chatsworth, CA, USA) with on-column DNAse I digestion (Qiagen), according to standard protocol. Quantitative and qualitative RNA analyses were performed using NanoDrop 1000 spectrophotometer (NanoDrop, Wilmington, DE, USA) and the Agilent 2100 BioAnalyzer (Agilent technologies, Palo Alto, CA, USA), respectively. Samples with RNA integrity (RIN) number greater than 9.0 were used for microarray analyses.

### Quantitative real-time PCR (qPCR) analysis

Complementary DNA (cDNA) was synthesized from 1 μg of total RNA using Superscript III (Invitrogen) according to standard protocol. qPCR was performed in 7500 Fast Real-Time PCR System (Applied Biosystems, Foster City, CA, USA) with Fast SYBR-Green Master mix (Applied Biosystems) according to the manufacturer’s protocol. Data for MDA-MB-231 represents triplicate experiments, while data for the other three cell lines represent one experiment. Triplicate technical replicates were used for each experiment. Primers were designed using NCBI Primer BLAST and evaluated for secondary structures using the OligoAnalyzer tool (IDT, Coralvile, NA, USA). All primers were obtained from IDT and their target specificity was verified with melting curve analysis. Primer sequences are available upon request. The ΔΔCt method was used to determine differential gene expression using 36b4 (RPLP0), 18S (from RNA45S5), or GAPDH as reference genes. The two-tailed Student's t-test was used for statistical testing, and results were considered significant if p<0.05.

### Western blot analysis

Whole cell lysate was prepared using RIPA Buffer supplemented with Roche Complete Mini Protease Inhibitor Cocktail (Sigma Aldrich, St. Louis, MO, USA) and protein quantified using the Pierce 660 Protein Assay Kit (Thermo Fischer Scientific, Rockford, IL, USA), according to manufacturers’ protocols. Approximately 30 μg of total protein was loaded onto pre-cast 10% polyacrylamide gels (Bio-Rad, Hercules, CA, USA) for SDS electrophoresis. Subsequently, proteins were transferred to nitrocellulose membranes (Millipore, Billerican, MA, USA), blocked with 10% non-fat milk in Tris-buffered saline Tween-20 (TBST), washed and probed with 1:800 rabbit polyclonal anti-MELK (Cell Signaling Technologies, Danvers, MA, CN: 2274), 1:1000 mouse monoclonal anti-MELK (SAB4300692, Sigma-Aldrich), 1:1000 mouse monoclonal anti-p53 (sc-126, Santa Cruz Biotechnologies, Dallas, TX), 1:1000 rabbit polyclonal anti-PARP (9542, Cell Signaling) or 1:6000 mouse monoclonal anti-β-actin (A2228, Sigma Aldrich) primary antibodies in 1% non-fat milk TBST overnight at 4°C. The washed membranes were probed with 1:2000 species-specific secondary antibodies conjugated with horseradish peroxidase (GE Healthcare, Little Chalfont, UK) for 2h at room temperature. After incubating in Western Bright ECL chemiluminescent substrate (ThermoFisher Scientific), the membranes were exposed to x-ray film and developed. Densitometric analysis (ImageJ software version 10.2) was used for quantification. OTS167-treated cells were analyzed after 48 or 92h.

### Microarray and data analysis

Spotted 70-mer arrays covering 36,000 genes and variants (full protein-coding genome) were used (Human Genome OpArray, Microarray, Inc., Huntsville, AL, USA), as previously described [17], and platform information, data and protocols is available at NCBI GEO (accession number [GSE68693]). RNA isolated after 48h and 72h treatments were analyzed in biological and technical duplicates. Slides were scanned using Gene Pix 4300A scanner and analyzed using GenePix Pro 6.0 software (Molecular Devices, Sunnyvale, CA, USA). R software (version 3.3.2) and the *limma* package [18] were used to filter and normalize data, perform dye-swaps, generate M values log2(fold change) for each slide, and perform statistical analyses (Bonferroni correction). Genes were considered differentially expressed if p-value < 0.05 and M>|0.4|). Pathway Studio software (Elselvier, Philadelphia, PA) was used to assess overrepresented gene ontologies and enriched transcription factor networks among differentially expressed genes for each cell line. p < .05 was considered significantly enriched.

### Analysis of clinical databases

Breast Cancer Gene-Expression Miner v4.0 (bc-GenExMiner v4.0) database [19] was used to evaluate expression in 5,349 clinical samples. Gene expression data was included from all available clinical tumors, comparing expression in 1,144 tumors classified as basal-like with 4,205 tumors classified as not basal-like. Box and whiskers plots are displayed, along with Welch's tests, as detailed by the database.

## Results

### MELK is highly expressed in TNBC

**Fig 1:**
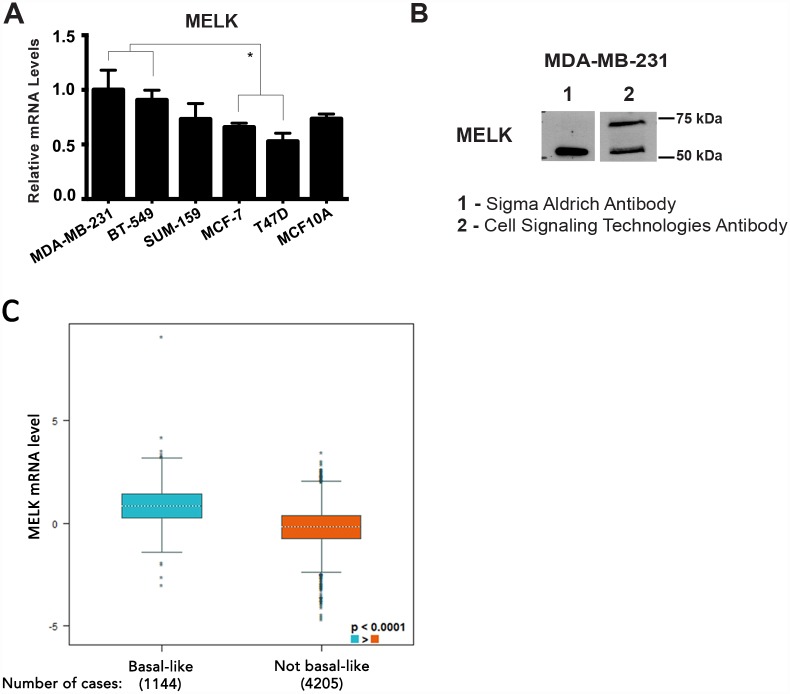
MELK is increased in TNBC cell lines and clinical samples. (A) MELK is upregulated in the TNBC cell lines MDA-MB-231 and BT-549 compared to the Luminal A subtypes MCF-7 and T47D. (B) Western blot using two different MELK antibodies. In lysates from MDA-MB-231 cells, both the Sigma Aldrich and Cell Signaling Technologies antibodies detect a band near 52 kDa, but only the latter antibody detects the proposed full-length MELK isoform near 74 kDa. (C) MELK expression in basal-like and/or TNBC tumors (n = 1,144) compared with not basal-like tumors (n = 4,205). MELK mRNA level showed a significant increase in basal-like/TNBC tumors, p-value (Welch's) < 0.0001.

We first confirmed the expression of MELK mRNA in six breast cell lines (characteristics listed in Table 1), including three TNBC claudin-low subtype cell lines (MDA-MB-231, BT-549 and SUM-159), two Luminal A (MCF-7 and T47D), and the non-tumorigenic MCF10A. We confirmed that MELK mRNA expression was significantly higher in the MDA-MB-231 and BT-549 TNBC cell lines compared to the Luminal A cell lines (Fig 1A), confirming previous reports [11, 20]. After applying two different anti-MELK antibodies towards protein isolated from MDA-MB-231 cells, we found that one of them (from Cell Signaling Technologies) detected two bands (74kDa and 52kDa), while the other (from Sigma-Aldrich) detected only the smaller band (Fig 1B). Since the full-length isoform of MELK is 74kDa, we decided to use the Cell Signaling polyclonal antibody, which has been applied in MELK studies previously [21], for the remainder of our study. Using the data available on MELK isoforms, we hypothesize that the smaller band detected by both antibodies is the 52kDa splice variant of MELK. We further used the Breast Cancer Gene-Expression Miner to evaluate MELK mRNA levels in clinical samples. Comparing MELK expression from basal-like tumors (n = 1,144) with not basal-like tumor (n = 4,205), we confirm significantly increased MELK expression in the basal-like BC cohort (p<0.0001; Fig 1C). This supports previously published data generated from 392 BC samples in the TCGA BC collection [11].

**Table 1:** Molecular subtype, p53 mutational status and other key mutations of breast cancer cell lines used in this study.

Cell Line Name	Molecular Subtype	p53 Status	Mutations
MDA-MB-231	TNBC / Claudin Low	mt R280K	KRAS, BRAF, CDKN2A
BT-549	TNBC / Claudin Low	mt R249S	PTEN, RB1
SUM-159	TNBC / Claudin Low	mt R158InF	HRAS, PIK3CA
T-47D	ER+ / Luminal A	mt L194F	PIK3CA
MCF-7	ER+ / Luminal A	WT	PIK3CA, CDK2NA
MCF-10A	Non-cancerous	WT	

### OTS167 reduces proliferation of TNBC cells and impacts MELK mRNA levels

**Fig 2:**
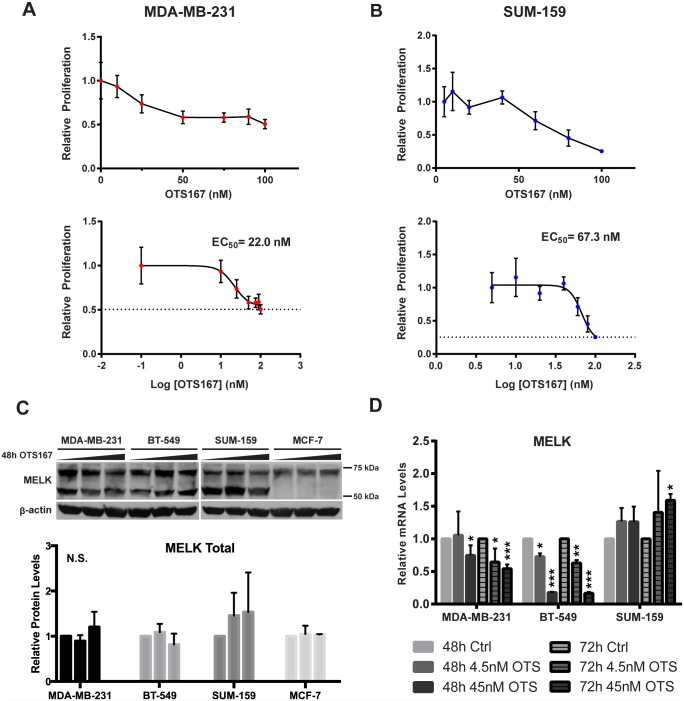
OTS167 reduces proliferation and regulates MELK transcript but not protein levels in TNBC cells. MTS assays were performed in (A) SUM-159 and (B) MDA-MB-231 cells treated with 5 nM to 100 nM OTS167 for 48h. EC_50_ values were calculated using the Nonlinear Regression (Curve Fit) function. Western blots revealed that OTS167 did not significantly alter MELK protein levels in the TNBC cell lines or MCF-7 following 48h treatment (densitometric data from two experiments). (C) The MELK 52 kDa variant was highly expressed in the TNBC cell lines but not in luminal A MCF-7. (D) qPCR demonstrated that MELK transcripts were downregulated by OTS167 in MDA-MB-231 and BT-549, but upregulated in SUM-159 after 48h and 72h. Significance was calculated using the Student’s two-tailed t-test: p < 0.05 (*), p < 0.01 (**), and p < 0.001 (***).

To explore the pharmacological inhibition of MELK *in vitro*, we focused on the kinase inhibitor OTS167 which is currently in clinical trials [22]. OTS167 has been reported to inhibit MELK with an EC_50_ of 8 nM—70 nM depending on the cell line [15]. To determine and validate the effective dose for use in our study, we performed MTS assays in MDA-MB-231 and SUM-159 with increasing concentrations of OTS167. Using these assays, we concluded that OTS167 had EC_50_ values of 22.0 nM in MDA-MB-231 (Fig 2A) and 67.3 nM in SUM-159 (Fig 2B). These doses are 10-fold higher than what has been found previously [15]. For the remainder of our studies, we used the average of the EC_50_ values from MDA-MB-231 and SUM-159 (45 nM) as well as a ten-fold lower concentration (4.5 nM) to study potential low-dose effects.

As MELK is stabilized via autophosphorylation [23], we hypothesized that its competitive inhibitor OTS167 would lead to its degradation. Following 48h treatment with OTS167, we did not find a significant reduction of total MELK protein levels (52 kDa and 74 kDa) in any of the cell lines tested, although a trend of decreasing levels appear in MDA-MB-231 and SUM159 cells (Fig 2C). Incidentally, we noted that the 52 kDa band was present in all three TNBC cell lines but not detected in Luminal A MCF-7 cells (Fig 2C). MELK transcripts, however, were significantly reduced in a dose-dependent manner in MDA-MB-231 and BT549, but not in SUM-159 (Fig 2D). Performing siRNA of MELK, we noted that the mRNA levels were reduced but the protein levels remained stable [data not shown]. We concluded that there were transcriptional feedback effects of the inhibitor and that these effects were prominent in MDA-MB-231 and BT-549 cells, but that the protein appeared stable and did not show reduced levels within 72h.

### OTS167 induces context-specific transcriptomic changes but common biological functions in MDA-MB-231 and SUM-159

**Fig 3:**
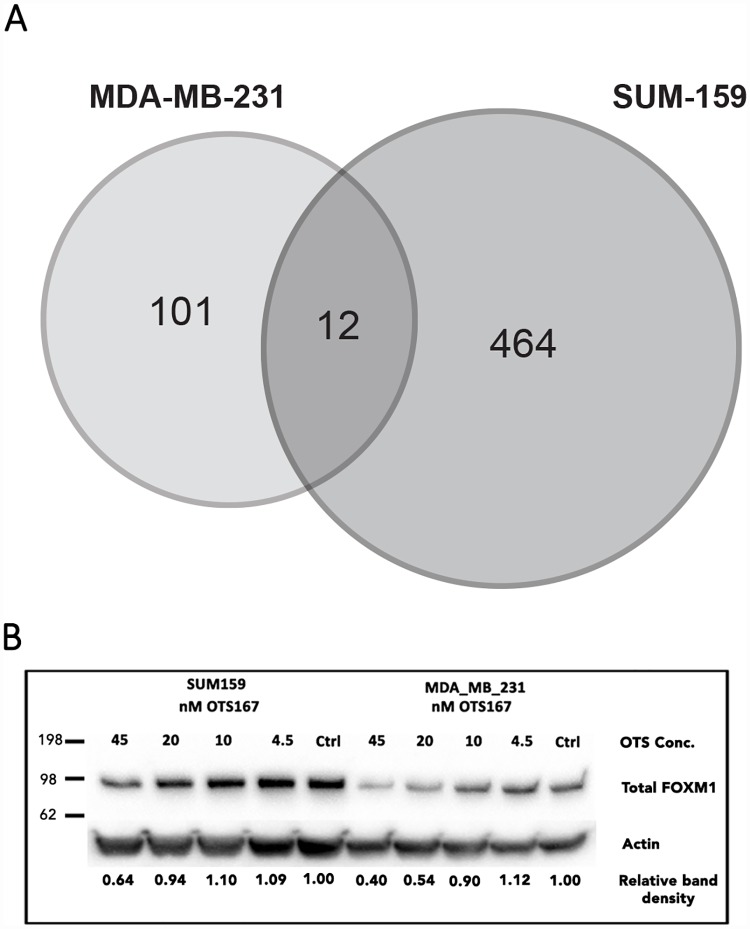
Differentially expressed genes identified by microarrays. A) Graphical representation of significantly regulated genes in MDA-MB-231 compared with SUM-159 cells after treatment with 45 nM OTS167 for 72h. Genes were considered regulated if p<0.05 and M > |0.4|. B) FOXM1 was identified as an enriched transcriptional regulator of the differentially expressed genes. FOXM1 total protein level was confirmed to decrease significantly upon OTS167 treatments. Cells were treated with different concentrations of OTS167 for 48h, and 20nM of OTS167 was sufficient to decrease FOXM1 protein levels in MDA-MB-231 and 45 nM in SUM-159 cells. Actin used a loading control, and densitometric data for relative band density is noted below.

Since MELK is anticipated to modify the phosphorylation state and thereby activity of several transcription factors, we performed microarrays to explore the transcriptomic effects of OTS167 and to identify its impact on corresponding signaling pathways. After treating SUM-159 and MDA-MB-231 with 4.5 nM and 45 nM OTS167 for 72h, RNA was extracted and microarray analysis performed [GEO accession #GSE68693]. At the lower concentration, 4.5 nM, few genes were detected as significantly regulated (data not shown) and thus, the remainder of the analysis focused on the 45 nM treatments. Treatment with 45 nM OTS167 resulted in 101 significantly regulated genes in MDA-MB-231 and 464 in SUM-159, with only 12 genes regulated in both cell lines (Fig 3A). Next, we explored which types of biological processes were affected by OTS167 in each cell line, searching for enriched Gene Ontology (GO) biological processes among all the upregulated (Table 2) and downregulated (Table 3) genes, respectively. We found that while many genes were regulated differently between the cell lines, were involved in same biological processes. Among upregulated genes in both cell lines, processes including mitosis/cell cycle/cell division, cellular response to DNA damage stimulus/DNA repair, apoptotic process and chromatin modification were enriched. Among downregulated genes, we found enrichment of cellular protein metabolic process, tRNA aminoacylation, protein transport, protein folding, small molecule metabolic process and angiogenesis functions. Thus, the microarray analysis identifies which biological functions are affected, and supports that the inhibitor mediates similar functions in both cell lines.

**Table 2:** Biological processes enriched among OTS167 upregulated genes.

GO Bioprocess	Genes	p-value
**MDA-MB-231/SUM-159**		
Mitosis	11/8	2.71E-05/ 1.29E-05
Cellular response to DNA damage stimulus	10/6	1.79E-03/ 4.05E-03
Cell Cycle	13/13	2.00E-03/ 5.47E-07
Cell Division	8/8	9.00E-03/ 5.56E-05
DNA repair	8/8	9.87E-03/ 6.24E-05
Apoptotic process	13/7	1.60E-02/ 3.27E-02
Chromatin modification	6/4	2.15E-02/ 1.73E-02
**MDA-MB-231 Only**		
Transcription, DNA-templated	40	5.88E-06
Neg. regulation of cell-matrix adhesion	4	6.31E-06
Neg. regulation of transcription from RNA poly II promoter	4	4.77E-04
Neg. regulation of transcription, DNA-templated	13	4.96E-04
Positive regulation of protein ubiquination	4	9.35E-04
Positive regulation of calcium ion import	2	3.37E-03
Phosphorylation	13	4.25E-03
Regulation of cell differentiation	5	7.60E-03
DNA recombination	4	1.06E-02
**SUM-159 Only**		
Nucleosome assembly	9	6.41E-09
G1-S transition of mitotic cell cycle	7	3.03E-06
DNA replication	6	5.28E-05
Telomere maintenance via recombination	3	1.19E-04
Nuclear migration	2	3.73E-04
Cytokine-mediated signaling pathway	5	2.42E-03
mRNA processing	5	8.12E-03
RNA splicing	4	2.82E-02
Intracellular signal transduction	5	3.63E-02

Gene lists of differentially expressed genes from MDA-MB-231 and SUM-159 after 72h treatment (45nM) were analyzed separately. Corresponding biological processes, as classified by Gene Ontology, that were significantly enriched in both cell lines are listed at the top; along with number of genes regulated in each cell lines and corresponding p-value, respectively. Processes enriched in only one of the cell line listed below.

**Table 3:** Biological processes enriched among OTS167 downregulated genes.

GO Bioprocess	Genes	p-Value
**MDA-MB-231/SUM-159**		
Cellular protein metabolic process	18/7	2.37E-06/ 7.19E-04
tRNA aminoacylation	3/1	1.74E-04/ 3.42E-02
Protein transport	16/5	3.77E-04/ 3.64E-02
Protein folding	6/5	9.48E-03/ 2.18E-04
Small molecule metabolic process	26/11	7.19E-04/ 1.95E-03
Angiogenesis	6/3	3.19E-02/ 3.56E-02
**MDA-MB-231 Only**		
Gene expression	26	1.47E-09
RNA metabolic process	15	1.76E-08
Apoptotic process	22	1.27E-05
Translational initiation/ elongation/ termination	9	1.29E-05
Tissue regeneration	4	2.60E-04
Glutathione metabolic process	4	1.60E-03
RNA splicing	9	2.99E-03
Ras signal transduction	4	6.33E-03
Protein stabilization	4	7.75E-03
**SUM-159 Only**		
Cellular amino acid biosynthetic process	4	3.50E-06
ER unfolded protein response	4	1.08E-04
Platelet activation	5	3.62E-04
Response to ER stress	3	3.65E-04
Cell redox homeostasis	3	7.75E-04
ECM organization	5	1.51E-03
Cell migration	4	1.66E-03
Wound healing	3	5.23E-03
Innate Immune Response	6	1.27E-02

Gene lists of differentially expressed genes from MDA-MB-231 and SUM-159 after 72h treatment (45nM) were analyzed separately. Corresponding biological processes, as classified by Gene Ontology, that were significantly enriched in both cell lines are listed at the top; along with number of genes regulated in each cell lines and corresponding p-value, respectively. Processes enriched in only one cell line listed below.

### Microarray analysis indicates that effects are mediated via transcription factors B-MYB and FOXM1

To further understand the potential mechanisms of OTS167, we explored predicted expression regulators of the upregulated (Table 4) and downregulated (Table 5) genes for each cell line separately. We identified B-MYB (MYBL2, v-myb avian Myeloblastosis viral oncogene homolog-like 2) and FOXM1 (Forkhead Box M1) as transcription factors whose targets were differentially expressed upon treatment with OTS167. Previous research has demonstrated that FOXM1 phosphorylation is influenced by MELK [24]. Thus, we propose that OTS167 modifies activity of B-MYB and FOXM1. We further explored the expression of FOXM1 after OTS167 treatment and as demonstrated in Fig 3B, total FOXM1 protein levels are decreased upon 20 nM and 45 nM 48h-OTS167 treatments in both MDA-MB-231 and SUM-159, which confirmed our hypothesis.

**Table 4:** Expression regulators of OTS167-upregulated genes.

Name	Symbol	Genes	p-Value
**MDA-MB-231/SUM-159**			
CCAAT Factors	-	9/6	4.38E-04/ 8.63E-04
B-Cell CLL/Lymphoma 2	BCL2	3/5	9.97E-03/ 4.65E-04
**MDA-MB-231 Only**			
Androgen Receptor	AR	11	1.12E-03
Myeloblastosis viral oncogene homolog	MYB	7	1.75E-03
Histone deacetylase 1	HDAC1	7	5.24E-03
Heat Shock 70kDa Protein 5	HSPA5	4	5.92E-03
Cyclin-dependent kinase inhibitor family	CDKN	3	6.38E-03
Mitogen-activated protein kinase kinase 1	MAP2K1	7	8.53E-03
Telomerase reverse transcriptase	TERT	4	1.02E-02
cAMP responsive element binding protein 1	CREB1	14	1.13E-02
SRC proto-oncogene, non-receptor RTK	SRC	8	1.82E-02
**SUM-159 Only**			
Retinoblastoma-Associated Protein family	E2F	8	1.08E-04
Kirsten Rat Sarcoma Viral Oncogene Homolog	KRAS	6	1.35E-04
Erb-B2 Receptor Tyrosine Kinase 2	ERBB2	6	1.62E-04
Avian Myelocytomatosis Viral Oncogene Homolog	MYC	10	4.07E-04
Forkhead Box M1	FOXM1	6	6.16E-04
Retinoblastoma 1	RB1	5	9.92E-04
NF-Kappa-B P65 Subunit	RELA	7	1.00E-03
Tumor Protein p53	TP53	12	1.66E-03
Retinoblastoma-Associated Protein 1	E2F1	7	1.72E-03

Transcription factors whose targets were enriched among upregulated genes (72h, 45nM) in MDA-MB-231 and SUM-159, respectively. Regulators enriched in both cell lines listed on top, along with target genes differentially expressed per each cell line and corresponding p-values. Regulators with targets significantly enriched in only one cell line are listed below.

**Table 5:** Expression regulators of OTS167-downregulated genes.

Name	Symbol	Genes	p-Value
**MDA-MB-231/SUM-159**			
SMAD family member 2	SMAD2	7/5	1.28E-02/ 1.77E-03
Eukaryotic translation initiation factor 2, subunit 1α	EIF2S1	4/3	1.62E-02/ 4.74E-03
**MDA-MB-231 Only**			
Proteasome endopeptidase complex	-	24	1.94E-06
SMAD family member 3	SMAD3	9	1.29E-05
SMAD family member 4	SMAD4	9	6.10E-05
Noggin	NOG	8	9.52E-05
Wingless-type MMTV integration family member 2	WNT2	4	7.50E-04
Mitogen-activated protein kinase family	MAPK	22	2.68E-03
Tumor protein p53	TP53	21	4.63E-03
Phosphatase and tensin homolog	PTEN	8	5.29E-03
Epidermal growth factor	EGF	18	5.30E-03
Transforming growth factor β1	TGFB1	30	8.43E-03
Nuclear factor κB	NF-κB	25	9.88E-03
Insulin-like growth factor 1	IGF1	16	1.08E-02
**SUM-159 Only**			
Activating transcription factor 6	ATF6	7	2.22E-08
Activating transcription factor 4	ATF4	7	3.40E-06
Avian Myelocytomatosis viral oncogene homologue	MYC	10	1.28E-04
Nitric oxide synthase 2	NOS2	5	3.32E-04
Platelet-derived growth factor β	PDGFB	3	1.02E-03
Bone morphogenetic protein 3	BMP3	2	1.26E-03
Early growth response 1	EGR1	6	4.41E-03
Brain-derived neurotrophic factor	BDNF	6	8.02E-03

Transcription factors whose targets were enriched among downregulated genes (72h, 45nM) in MDA-MB-231 and SUM-159, respectively. Regulators enriched in both cell lines listed on top, along with target genes differentially expressed per each cell line and corresponding p-values. Regulators with targets significantly enriched in only one cell line are listed below.

### qPCR confirms differentially expressed genes

**Fig 4:**
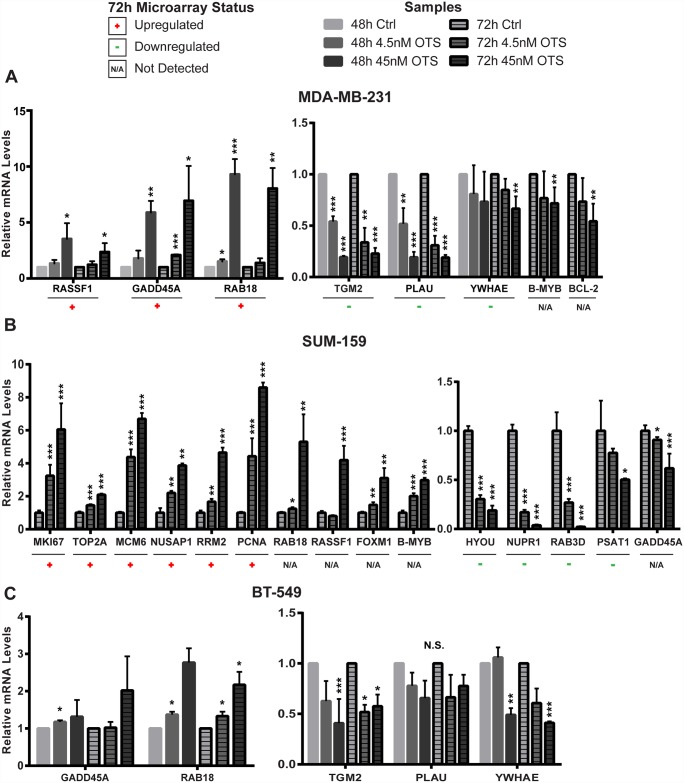
qPCR confirms gene expression changes identified in the microarrays. (A) MDA-MB-231, (B) SUM-159, and (C) BT-549 after 48h and 72h treatments of 45 nM and 4.5 nM OTS167. Significance was calculated using the Student’s two-tailed t-test: p < 0.05 (*), p < 0.01 (**), and p < 0.001 (***). Data for MDA-MB-231 consists of three experiments, data for SUM-159 represents one experiment and data for BT-549 represents two experiments. All experiments were performed in triplicates.

To confirm our microarray data, we randomly selected ten genes identified as differentially expressed in MDA-MB-231 and SUM-159 for further qPCR analysis, and also assessed the expression of selected genes in treated BT-549 cells. We confirmed the microarray data for 6/10 genes in MDA-MB-231 (Fig 4A) and 10/10 for SUM-159 (Fig 4B). Further, we found that although RAB18 (Ras-related small GTPase 18) and RASSF1 (Ras-association domain family member) were only detected as upregulated in the MDA-MB-231 microarray, they were also significantly upregulated in SUM-159 (Fig 4A and 4B). Additionally, TGM2 (transglutaminase 2), YWHAE (tyrosine 3-monooxygenase/ tryptophan 5-monooxygenase activation protein, epsilon) and GADD45A (growth arrest and DNA-damage-inducible, alpha), were identified as regulated in the MDA-MB-231 microarray, and were similarily regulated in BT-549 cells (Fig 4A and 4C). In SUM-159, however, GADD45A was regulated in the opposite direction (Fig 4B).

Although not identified as changed in the microarray analysis, we also included B-MYB and FOXM1 on the basis of their indications of target genes’ regulators (Table 4), as described above. We found that upon OTS167 treatment, B-MYB and FOXM1 transcripts were upregulated in SUM-159, while B-MYB was downregulated in MDA-MB-231 (Fig 4A and 4B). Thus, although FOXM1 protein was reduced upon treatment in both cell lines (Fig 3B), its transcript was upregulated in SUM-159. Taken together, OTS167 treatment resulted in different patterns of gene expression in the different TNBC cell lines, with some overlaps, and one gene, RAB18, was consistently upregulated in a dose-dependent manner in all three.

### OTS167 influences cell morphology, proliferation and apoptosis

**Fig 5:**
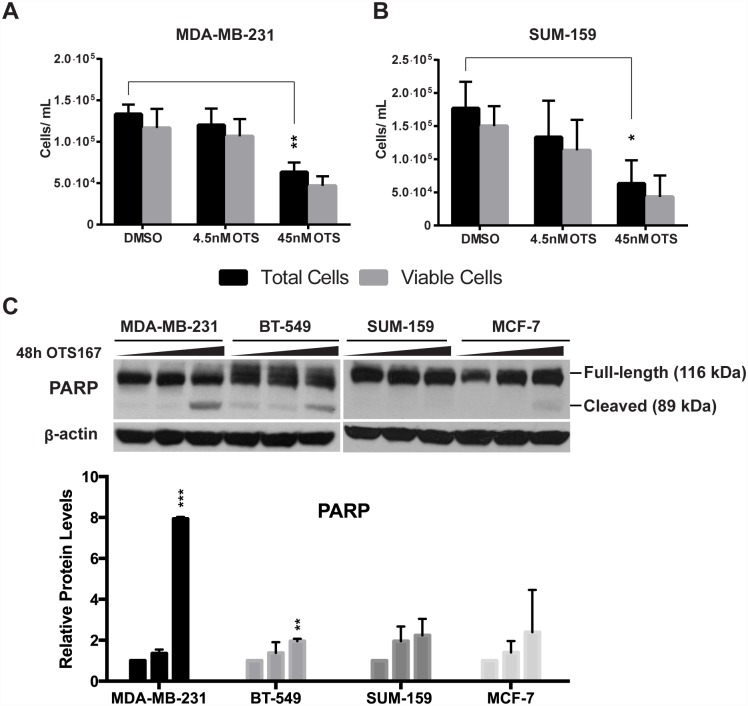
OTS167 treatment reduces total cell number and induces apoptosis. 45 nM treatments in (A) MDA-MB-231 and (B) SUM-159 significantly reduces total cell number but not the ratio of viable cells, as measured using trypan blue assay after 72h. (C) Apoptosis was identified by PARP cleavage after 48h treatments with either 4.5 nM or 45 nM OTS167. PARP was significantly cleaved in MDA-MB-231 and BT-549, but not in SUM-159 after 48h 45 nM OTS167 treatment (densitometric data from two experiments). MCF-7 displayed faint PARP cleavage after 45 nM treatment, but also an increase in full-length PARP. Significance was calculated using the Student’s two-tailed t-test: p < 0.05 (*), p < 0.01 (**).

Many of the genes regulated upon OTS167 treatment were enriched within proliferation, apoptosis and morphology. Proliferation has previously been reported to be reduced by OTS167 [15] and did so also in our experiment (Fig 2A and 2B). Using trypan blue exclusion assay, we confirmed that 45 nM OTS167 significantly reduced total cell number relative to control in MDA-MB-231 (Fig 5A) and SUM-159 (Fig 5B), with no significant difference in the ratio of live/dead cells. Using western blot, however, we identified significant PARP cleavage upon treatment with OTS167 (45 nM, 48h) in MDA-MB-231 and BT-549, but not in SUM-159 (Fig 5C). In MCF-7 cells, we observed low levels of PARP cleavage (N.S.), but noted an increase of full-length PARP (Fig 5C).

**Fig 6:**
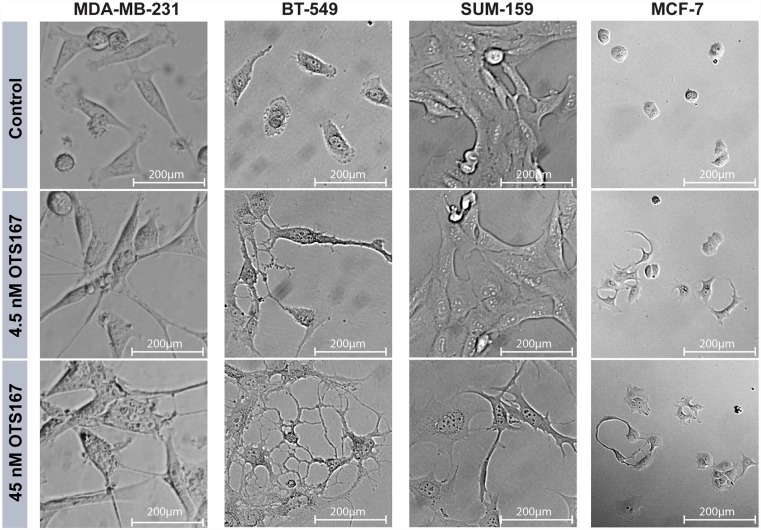
OTS167 results in morphological changes. MDA-MB-231, BT-549, SUM-159 and MCF-7 cells imaged after 24h treatment. The morphological changes are evident in each cell line tested after 45 nM OTS167 concentration, and in three cell lines (not SUM-159) at 4.5 nM. Phase-contrast images were taken at 40x magnification.

Additionally, we noticed drastic morphological changes in all three TNBC cell lines as well as in the MCF-7 luminal A cell line upon treatment with OTS167, (Fig 6). Within 24h following 45 nM treatments, it appeared as if cells had developed dendritic processes and/or were losing focal adhesions. These changes were also evident with the lower (4.5 nM) OTS167 concentration in all cell lines except SUM-159. Together, the reduced cell number, increased apoptosis, and morphological changes suggest that OTS167 has clear effects on several major signaling pathways identified in the microarray.

### OTS167 treatment decreases mutant p53 protein

**Fig 7:**
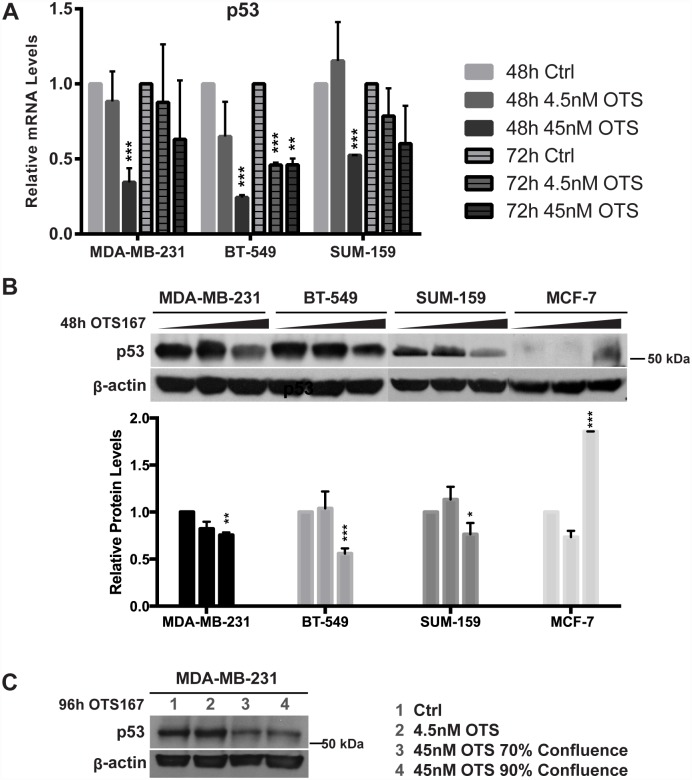
OTS167 reduces transcript and protein levels of mutant p53. (A) qPCR reveals that mutant p53 transcript is significantly downregulated in MDA-MB-231, BT-549 and SUM-159 after 48h and 72h (45 nM) OTS167 treatment. (B) Western blots show that mutant p53 protein is significantly reduced in MDA-MB-231, BT-549 and SUM-159, but WT p53 is increased in MCF-7 (48h, 45 nM OTS167, densitometric data from three experiments). (C) p53 protein reduction is independent of cell density (MDA-MB-231, 96h, 45nM OTS167). Significance was calculated using a Student’s two-tailed t-test: p < 0.05 (*), p < 0.01 (**), p < 0.001 (***).

After analyzing the microarray data for enriched expression regulators, we identified p53 (TP53) as regulator of upregulated genes in SUM-159 and of downregulated genes in MDA-MB-231 (Tables 4 and 5). Since p53 is disproportionately mutated in TNBC relative to other BCs, we evaluated its expression and regulation by OTS167 at both mRNA and protein level. As described previously, p53 is mutated in MDA-MB-231, SUM-159 and BT-549, but wild type (WT) in MCF-7 (Table 1). We found that mutant p53 transcripts were significantly downregulated in all three TNBC cell lines upon treatment with 45nM OTS167 for 48h (Fig 7A). Correspondingly, mutant p53 protein decreased significantly in all three TNBC cell lines after this treatment (Fig 7B). In MCF-7 cells, however, the same treatment resulted in an increase of WT p53 protein. As a control, we performed a western blot of MDA-MB-231 cells at a later time point (96h) and at different confluences (70% and 90%). Again, we found that OTS167 decreased mutant p53 protein levels and that this was independent of cell density (Fig 7C). These observations indicate that OTS167 treatment downregulates mutant p53 but upregulates WT p53 in BC.

## Discussion

Kinases can phosphorylate transcription factors and thereby modulate their activity. The goal of our study was to explore the underlying mechanisms of the MELK kinase inhibitor OTS167 in claudin-low TNBC cells. Microarray analyses of MDA-MB-231 and SUM-159 cells after treatment with 45 nM OTS167 or vehicle identified primarily different gene expression patterns in the two cell lines. However, genes with functions in processes such as cell cycle and apoptosis were overrepresented in both gene expression profiles. Since transcription factors are expressed at varying levels in different cell lines and may have cell-specific activity, the same genes are not necessarily expected to be regulated following OTS167 treatment. Our data indicate several common transcription factors whose activity changes pointing towards a general mechanism whereby OTS167 and MELK affect claudin-low TNBC.

From the microarray analysis, we identified p53 as a potential common upstream regulator of OTS167 effects. MDA-MB-231, SUM-159 and BT-549 all harbor homozygous mutant p53 [25, 26], and we found that OTS167 decreased its levels in all. WT p53 in the luminal A MCF-7 cell line, however, was increased upon the same treatment. Several investigators have proposed that MELK is involved in the function of p53, and MELK has been reported to decrease WT p53 in glioblastoma U87 cells [27]. Our finding that MELK inhibition significantly decreased mutated p53, both at the transcriptional and protein level, is novel and could have a clinical impact. Mutated p53 protein is more stable than WT, and while being unable to act as a tumor suppressor, it can exhibit gain-of-function characteristics. Many studies have proposed a functional link between CSCs and mutated p53 [28]. Contrary to WT p53, which is known to control stem cell populations and induce differentiation, mutated p53 can upregulate drug resistance pathways and has been correlated with stem cell transcriptional signatures in breast cancer [28, 29]. When mutant p53 is silenced in TNBC, this results in apoptosis and reduced expression of pro-survival genes [30–32]. As discussed previously, the claudin-low breast cancer cell subpopulation has CSC characteristics and is disproportionally mutated in p53. Since CSCs have been found to be disproportionately resistant to conventional cancer therapies and are hypothesized to contribute to cancer recurrence [33], one potential approach to eliminate these cells in therapy is to target the pathways responsible for their phenotype. Our data support that MELK inhibition may be useful in this respect.

We also found that OTS167 affects several other genes that may be linked to the claudin-low phenotype. The highly conserved RabGTPase RAB18 was significantly upregulated in MDA-MB-231, SUM-159 and BT-549 upon treatment. This is in agreement with the increase of RAB18 transcripts observed as Melk decreased during mouse mammary stem cell differentiation [12] Accordingly, we observed that differentiation was a process enriched amongst upregulated genes from the MDA-MB-231 microarray upon MELK inhibition (Table 2). Another gene, TGM2, was significantly and dose-dependently downregulated by OTS167 in MDA-MB-231 and BT-549 cells. TGM2 promotes EMT and treatment resistance and is a potential target for CSC populations. Silencing of TGM2 in MDA-MB-231 has been shown to reverse EMT [34] and lower expression of TGM2 is associated with increased sensitivity to chemotherapeutic agents in breast cancer [34, 35]. We also noted significant decreases in PLAU expression in MDA-MB-231 cells. Like TGM2, PLAU is a known mediator of metastasis and invasion and is highly expressed in several TNBC cell lines, including MDA-MB-231 [36, 37]. Thus, it is possible that OTS167 treatment results in reduced stemness of TNBC cells through decrease of mutated p53, TGM2 and PLAU levels, and upregulation of RAB18.

Changes in morphology were noted in all cells upon OTS167 treatment (Fig 5). Our observations correlate well with the cytoskeletal changes resulting from MELK knockdown in gastric cancer [38] and glioblastoma cells [39], and from OTS167 treatment in non small-cell lung cancer [40]. Since OTS167 treatment reduces cell number, stress due to dysregulation of focal adhesions can result in a similar phenomenon as anoikis, from which the cell cannot resolve and eventually leads to apoptosis. Further research could confirm whether these morphological changes align with processes such as differentiation and/or senescence as a result of treating cells with OTS167.

We found some divergent functional effects upon OTS167 treatment in MDA-MB-231 and SUM-159. While OTS167 reduced cell proliferation in both cell lines, SUM-159 cells had a higher EC_50_, no significant PARP cleavage, and a different gene signature upon treatment compared to MDA-MB-231. Levels of MELK transcripts were lower in SUM-159 compared to MDA-MB-231 and BT-549 (Fig 1), which possibly explains why its inhibition generated weaker effects on apoptosis and proliferation. Since we found that apoptosis was a biological process significantly enriched for among upregulated genes in the SUM-159 microarray (Table 2), it is possible that SUM-159 was undergoing caspase-independent apoptosis or that the concentration of OTS167 was too low to induce PARP cleavage.

Additionally, there is evidence that SUM-159 is more resistant to OTS167 relative to MDA-MB-231 and BT-549. For example, the MELK transcript was upregulated in response to OTS167 treatment in SUM-159 cells, while it was repressed in both MDA-MB-231 and BT-536 (Fig 2C). In addition to p53 we found an alteration in FOXM1-linked pathways. FOXM1 is a transcription factor known to upregulate MELK in basal-like breast cancers [11] and MELK inhibition has been reported to downregulate FOXM1 in other cancers [40, 41]. We hypothesized a FOXM1-MELK positive-feedback mechanism, where MELK stabilizes FOXM1 and FOXM1 induces MELK expression. We then found that OTS167 treatment decreases FOXM1 total protein levels in both SUM-159 and MBA-MD-231 cells, which supports this hypothesis. As shown in Fig 3B, this decrease appeared stronger and occurred at lower concentrations in MDA-MB-231 cells (20 nM) than in SUM-159 cells (45 nM,). It is possible that the upregulation of FOXM1 transcript upon OTS167 treatment noted in SUM-159 cells only opposes the reduction of the protein levels, contributing the decreased sensitivity of these cells to treatment. FOXM1 is considered to be an important protein target in cancer diagnosis and therapy, and there are new drugs in the process of being developed that can modulate the function of FOXM1 in the context of carcinogenesis [42]. For this reason, the effect of OTS167 decreasing FOXM1 levels could be clinically significant.

We found that while OTS167 decreased mutant p53 in the TNBC cell lines, it dose-dependently upregulated GADD45A (a p53 target) in MDA-MB-231 and BT-549 while downregulating it in SUM-159. Studies have shown that GADD45A exhibits context-dependent effects, in that it stimulates proliferation in MYC-driven breast cancers but induces apoptosis and senescence in Ras-driven breast cancers [43]. Since MDA-MB-231 and SUM-159 have activating mutations in KRAS and HRAS respectively [44], it is possible that GADD45A could be functioning as a tumor suppressor in these cell lines. The fact that GADD45A is downregulated in SUM-159 as a result of OTS167 treatment and that it is presumably pro-apoptotic in this context could further explain why we did not observe PARP cleavage in these cells. Taken together, our observations suggest that the SUM-159 cell line is less sensitive to OTS167 and possibly resisting treatment through the upregulation of other signaling pathways. In SUM-159 we also observed the upregulation of several genes associated with the cell cycle which are frequently upregulated in TNBC and correlate with poor prognosis (including Ki67/MKI67 [45], TOP2A [46], MCM6 [47], NUSAP1 [48], RRM2 [49], and PCNA [50]). Although these genes bring the therapeutic efficacy of OTS167 into question in this context, other genes that correlate with poor prognosis in BC were downregulated (including HYOU [51], NUPR1 [52], RAB3D [53], and PSAT1 [54]). Despite the upregulation of several cell cycle genes in SUM-159, we noted a clear reduction of proliferation and cell number after OTS167 treatment. One possibility is that treatment with OTS167 induces cell cycle arrest. In fact, research in glioblastoma has demonstrated that silencing MELK induces G1/S cell cycle arrest in U87 cells, which is accompanied by a senescence-like phenotype [39]. Likewise, it has been found that a different MELK inhibitor (MELK-T1) decreases the proportion of cells in S phase relative to control while increasing their number in G1 in MCF-7 cells [55]. We detected only 113 genes as regulated in MDA-MB-231, which is a low number of genes. When fewer genes are regulated, there is a risk of more background noise being recorded as differential expression (false positives). We could not confirm all genes detected as regulated in the microarray with qPCR, and we conclude that a portion of the 113 genes is likely false positives.

As a result of the divergent effects in MDA-MB-231 and SUM-159, we were also concerned with the specificity of OTS167. OTS167 functions by competing for ATP binding in the active site of MELK [56]. Inhibitors targeting such highly-conserved regions of kinases are generally not highly selective in their mechanism of action and their efficacy relies on the abundance of the target relative to the other affected kinases [57, 58]. OTS167 at very high concentrations (10 μM) has been shown to decrease the activity of up to 210 kinases [59]. Since RNA-seq data indicate that several of these kinases are expressed at high levels in these two cell lines [60], it is plausible that some cell-specific effects identified in our study result from off-target effects rather than selective inhibition of MELK, even though the concentrations used in our study are far lower. For example, the kinase Aurora B is more highly expressed than MELK in these cell lines according to RNA-seq data [60], and has recently been confirmed as a target for OTS167 [61]. In our study the inhibitor appeared to induce more similar effects in BT-549 and MDA-MB-231, and future studies may explore the genome-wide effects in this cell line. Furthermore, the effect of OTS167 on wild-type p53 should be tested in other WT p53 breast cancer cell lines to establish whether it is similarly upregulated. Exploring additional time points and shorter intervals after treatment, would also be useful to identify how soon after treatment the transcriptional effects can be recorded. Possibly, this could help differentiate between direct and indirect effects. In our study, the concentrations required for the EC_50_ were higher than what has been reported previously [15]. This may be due to using OTS167 manufactured from different vendors, methodological considerations, or variability in cell line sensitivity in different labs.

In conclusion, we here describe the effect of the MELK inhibitor OTS167 in different claudin-low TNBC cells, and describe, for the first time, its genome-wide effects. The changes in gene expression identified by our microarray translate into biological processes affecting proliferation, DNA repair, apoptosis and angiogenesis, and point to specific transcription factors as key mediators. Specifically, we identify that OTS167 treatment in three different claudin-low TNBC cell lines leads to an upregulation of RAB18 and a downregulation of mutant p53.

We confirm reduction of proliferation and increase of apoptosis, along with morphological changes upon treatment with the inhibitor. We further propose that OTS167 might be operating in part through different signaling pathways in SUM-159 and MDA-MB-231, which could reflect context-dependent and/or off-target effects.
